# Species-Specific Effects on Ecosystem Functioning Can Be Altered by Interspecific Interactions

**DOI:** 10.1371/journal.pone.0165739

**Published:** 2016-11-03

**Authors:** David S. Clare, Matthew Spencer, Leonie A. Robinson, Christopher L. J. Frid

**Affiliations:** School of Environmental Sciences, University of Liverpool, Brownlow Street, Liverpool, L69 3GP, United Kingdom; Sveriges lantbruksuniversitet, SWEDEN

## Abstract

Biological assemblages are constantly undergoing change, with species being introduced, extirpated and experiencing shifts in their densities. Theory and experimentation suggest that the impacts of such change on ecosystem functioning should be predictable based on the biological traits of the species involved. However, interspecific interactions could alter how species affect functioning, with the strength and sign of interactions potentially depending on environmental context (e.g. homogenous *vs*. heterogeneous conditions) and the function considered. Here, we assessed how concurrent changes to the densities of two common marine benthic invertebrates, *Corophium volutator* and *Hediste diversicolor*, affected the ecological functions of organic matter consumption and benthic-pelagic nutrient flux. Complementary experiments were conducted within homogenous laboratory microcosms and naturally heterogeneous field plots. When the densities of the species were increased within microcosms, interspecific interactions enhanced effects on organic matter consumption and reduced effects on nutrient flux. Trait-based predictions of how each species would affect functioning were only consistently supported when the density of the other species was low. In field plots, increasing the density of either species had a positive effect on organic matter consumption (with no significant interspecific interactions) but no effect on nutrient flux. Our results indicate that species-specific effects on ecosystem functioning can be altered by interspecific interactions, which can be either facilitative (positive) or antagonistic (negative) depending on the function considered. The impacts of biodiversity change may therefore not be predictable based solely on the biological traits of the species involved. Possible explanations for why interactions were detected in microcosms but not in the field are discussed.

## Introduction

The impact of biodiversity change on the functioning of ecosystems is a pressing environmental concern [1,2]. An extensive body of research shows that the provision of key ecological functions, such as resource acquisition and nutrient retention, tends to decline with decreasing species richness [3,4]. Less attention, however, has been given to other aspects of biodiversity change, such as species turnover (i.e. species being replaced by other species) or shifts in the densities of constituent species. The impacts of these phenomena are pertinent given the degree and ubiquity with which they are currently occurring [5–7], with examples ranging from plankton [8] through to birds [9] and primates [10]. Greater insight into how biodiversity change, in its various forms, affects ecosystem functioning is therefore needed.

Experimental evidence suggests that the provision of ecological functions varies with respect to the presence and densities of species with particular biological traits [11,12]. In terrestrial plant assemblages, for example, carbon sequestration is driven by populations of slow-growing and long-lived species [13], whereas nutrient cycling is highly affected by nitrogen-fixers [14]. The impacts of biodiversity change on ecological function provision should therefore be predictable based on the traits of the species involved. However, interspecific interactions may also play an important role in ecosystem functioning, either through facilitation (i.e. positive interactions [15,16]) or antagonism (i.e. negative interactions [17,18]). Such interactions could amplify or weaken species-specific effects on function provision, respectively, thereby inhibiting our ability to use biological traits to predict changes to ecosystem functioning when species are extirpated, introduced or experience shifts in their densities. To date, a role of interspecific interactions in regulating ecosystem functioning has generally been demonstrated under controlled environmental conditions (but see [17]). The effects of interspecific interactions on function provision within natural systems is therefore particularly unclear. It is possible that such effects will be overshadowed [19,20], weakened [16,18] or reversed in sign [21] by the environmental heterogeneity encountered in nature.

The amphipod *Corophium volutator* and the polychaete *Hediste diversicolor* are ideal model organisms with which to investigate how species-specific effects on ecosystem functioning are influenced by interspecific interactions. Commonly found within intertidal soft sediments of the temperature North Atlantic, these species often dominate infaunal biomass [22–26] and are therefore likely to be major contributors to their associated ecological functions [27,28]. While co-occurrence is commonplace, negative correlations between the densities of *C*. *volutator* and *H*. *diversicolor* have been reported at multiple sites (e.g. [29–31]), as have temporal shifts in their dominance (e.g. [32]). The natural dynamics of *C*. *volutator* and *H*. *diversicolor* therefore make them particularly suitable for studying how ecosystem functioning varies under realistic shifts in the densities of species

**Fig 1:**
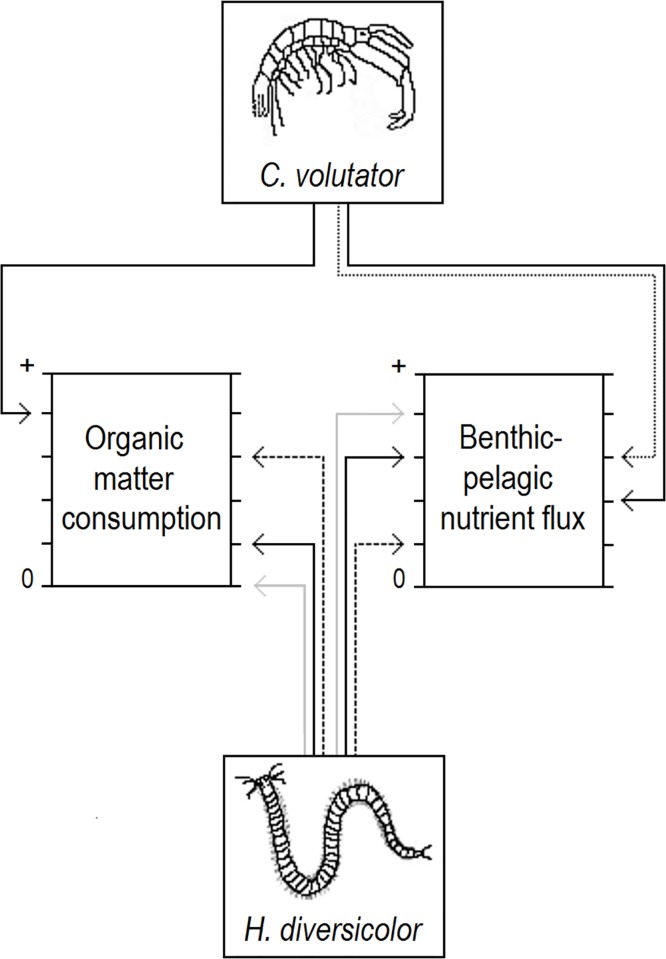
Illustration of hypotheses. The predicted effects that increasing the densities of *Corophium voluator* and *Hediste diversicolor* will have on organic matter consumption and benthic-pelagic nutrient flux, from 0 (no effect) toward an increasingly positive effect. The solid black lines represent predictions based on the biological traits of *C*. *volutator* and *H*. *diversicolor*. For *C*. *volutator*, the dotted line represents how an interaction with *H*. *diversicolor* may enhance its effect on nutrient flux. For *H*. *diversicolor*, the dashed lines represent how an interaction with *C*. *volutator* may enhance its effect on organic matter consumption and reduce its effect on nutrient flux; whereas the grey lines represent how a different interaction with *C*. *volutator* may reduce its effect on organic matter consumption and enhance its effect on nutrient flux.

In the present study, we investigated the effects of concurrent changes to the densities of *C*. *volutator* and *H*. *diversicolor* on organic matter consumption and benthic-pelagic nutrient flux (hereafter nutrient flux); key ecological functions that underpin waste assimilation and pelagic primary production, respectively. *C*. *volutator* constructs shallow burrows (< 5 cm) from which it feeds on organic deposits on the sediment surface [33,34], whereas *H*. *diversicolor* flushes relatively deep burrows (< 15 cm) as a means of suspension-feeding and obtaining oxygen [24,35]. It was therefore predicted that an increase in the density of *C*. *volutator* would lead to enhanced organic matter consumption and that an increase in *H*. *diversicolor* density would lead to enhanced nutrient flux (Fig 1, solid black lines). We note, however, that burrow construction by *C*. *volutator* is likely to cause a flux of nutrients from the upper sediment layers [36], and that this process may be enhanced at high densities of *H*. *diversicolor* due to burrow disturbance by the polychaete ([37], Fig 1, dotted line). Moreover, the trophically-plastic *H*. *diversicolor* can feed on deposits as well as suspended particles [24]. High densities of *C*. *volutator* could cause *H*. *diversicolor* to switch to deposit-feeding, as sediment resuspension by *C*. *volutator* [38] might reduce suspension-feeding efficiency (Fig 1, dashed lines), or feed more exclusively on suspended particles (thus flushing its burrow more regularly), so as to avoid interspecific competition with *C*. *volutator* (Fig 1, grey lines). We therefore postulated that the effects of the two species on ecosystem functioning might interact as their densities change, with the sign of the interactions possibly depending on the function considered. These hypotheses were tested using complementary experiments in laboratory microcosms and field plots, to determine whether any effects observed under homogenous conditions are also observed under natural, heterogeneous conditions

## Materials and Methods

The study consisted of two experiments: one conducted within laboratory microcosms and the other conducted *in situ* within an intertidal mudflat on the Mersey Estuary, Liverpool, UK (53° 21ʹ 22ʺ N, 02° 55ʹ 33ʺ W). Permission to extract samples was granted by the NW Inshore Fisheries Conservation Authority (shellfish) and Natural England.

Assemblages of various densities of *Corophium volutator* and *Hediste diversicolor* were created for both experiments. To assess the effects of the species on ecosystem functioning, the mass of consumed organic matter and nutrient concentrations were measured directly in the microcosms and by proxy in the field, due to methodological constraints. The experiments were run for 12 days, which roughly corresponds to the duration of most other studies that have assessed biotic influence over ecosystem functioning using benthic macroinfauna. The effects of macroinfaunal species on functioning are known to manifest within such short periods (e.g. [39,40]); however, the effects of interactions among species may become more prominent beyond this period [31]. Practical issues regarding the rapid depletion of organic detritus added to microcosms and difficulties in maintaining the desired species density treatments in field plots over time made a longer experiment duration unfeasible.

### Experimental procedure

#### Microcosm experiment

Sediment containing *C*. *volutator* and *H*. *diversicolor* was collected from the field study site and sieved through 0.5 mm mesh in a bath of seawater. Retained individuals of the two study species were incubated in aerated aquaria at the approximate temperature and salinity of the field site (15°C; 30 psu). Sieved sediment was captured, allowed to settle for 24 hours and homogenised after draining the overlying water. The alga *U*. *intestinalis* was also collected from the study site, rinsed with seawater and dried at 70°C to be used as organic detritus.

Microcosms (approximately 0.01 m^2^) were created in the laboratory by filling opaque cylindrical vessels (10 cm diameter, 40 cm height) with 0.75 l of homogenised sediment (10 cm depth) and 1.50 l of overlying water (30 psu; 20 cm depth), which had been pre-filtered through 1.2 μm mesh. After a settling period of 24 hours, the study species were transferred from aquaria into the vessels. After a further two days the water was replaced to remove excess nutrients associated with assembly and detritus was added to the vessels and allowed to settle onto the sediment surface. Microcosms were aerated throughout the 12-day experimental period (starting at macroinfauna addition) and kept at a constant temperature of 16°C under artificial lighting.

Ten experimental treatments were created within the microcosms. The first five represented a progressive shift from *C*. *volutator* (*Cor*) to *H*. *diversicolor* (*Hed*) dominance: 1) 1.00 g *Cor*, 0.00 g *Hed*; 2) 0.75 g *Cor*, 0.25 g *Hed*; 3) 0.50 g *Cor*, 0.50 g *Hed*; 4) 0.25 g *Cor*, 0.75 g *Hed*; 5) 0.00 g *Cor*, 1.00 g *Hed*. Initial total biomass was set to 1 g to match macroinfaunal density at the field site at the time of the experiment. Three additional treatments, reflecting an increase in *H*. *diversicolor* density while *C*. *volutator* density remained consistently high, were also included: 6) 1.00 g *Cor*, 0.25 g *Hed*; 7) 1.00 g *Cor*, 0.50 g *Hed*; 8) 1.00 g *Cor*; 1.00 g *Hed*. These treatments were used to aid comparisons between microcosm and field experiments, as incrementally increasing the density of *H*. *diversicolor* across field plots did not cause the dominant *C*. *volutator* to decline in density (i.e. the intended shift in species dominance was not achieved; see *Field experiment* within the ‘Experimental procedure‘ and ‘Data analysis’ sections of Materials and Methods). One gram of organic detritus (dried *U*. *intestinalis*) was added to each of the above treatments. Two control treatments were also used: 9) detritus addition but no fauna addition, and 10) no detritus addition and no fauna addition. These treatments provided a baseline from which to measure the effects of the study species and allowed the influence of detritus addition on nutrient concentration to be determined. Each treatment was replicated six times.

Duplicate samples of overlying water (5 ml) were taken at the end of the experiment and sediment contained within each microcosm was sieved through 0.5 mm mesh. Additional sieves of 0.355 and 0.235 mm mesh were stacked below to capture fragments of detrital *U*. *intestinalis* that passed through the 0.5 mm sieve. The wet biomasses of *C*. *volutator* and *H*. *diversicolor* were immediately weighed to provide comparable measurements to the start of the experiment. Retained *U*. *intestinalis* was dried at 70°C and re-weighed. Organic matter consumption was indicated by the loss of mass of detrital *U*. *intestinalis* (*sensu* [41]). Ammonium (NH_4_-N) and nitrate (NO_3_-N) concentrations in the water samples were measured using a Seal Analytical AutoAnalyser 3 HR, calibrated using Analar Grade solid ammonium sulphate and potassium nitrate dissolved to 100 ppm stock standards, and summed to give the dissolved inorganic nitrogen (DIN) concentration. Variation in DIN concentration among microcosms was taken to indicate variation in the level of nutrient flux.

Due to the large total number of microcosms (n = 60), the experiment was temporally staggered across six experimental runs, each consisting of a single block of ten microcosms (one replicate of each treatment). The experiment began on the 1^st^ of September 2014 and an experimental run was initiated every 7 days. Within each block, all microcosms were created using the same batch of homogenised sediment, seawater, macroinfauna and *U*. *intestinalis*.

#### Field experiment

At the time of the field experiment (June-July 2014), macroinfaunal biomass at the study site was dominated by *C*. *volutator* (mean density = 45 g m^-2^) followed by *H*. *diversicolor* (mean density = 10 g m^-2^). As previous studies suggest that elevated densities of *H*. *diversicolor* cause the density of *C*. *volutator* to decline [29,42], *H*. *diversicolor* was added to experimental plots (approximately 0.03 m^2^) across a range of densities (described below) with the aim of creating a progressive shift from *C*. *volutator* dominance to *H*. *diversicolor* dominance. Plots were not enclosed, so as to allow *C*. *volutator* to emigrate in response to the addition of *H*. *diversicolor* and to avoid introducing an experimental artefact.

To obtain individuals for transplantation, sediment containing *H*. *diversicolor* was collected from the study site and sieved through 0.5 mm mesh. Retained individuals were incubated as described above for the microcosm experiment. The various density treatments were weighed out in the laboratory and transported to the experiment site within plastic containers filled with water from the aquaria used for incubation. Individuals were transplanted into plots that were marked out by pressing a corer (20 cm diameter) onto the sediment surface. Small holes were created in the sediment (using a metal rod; 0.5 cm diameter) to encourage individuals to burrow within the plot area.

*H*. *diversicolor* was added to plots at densities of 1 g (32 g m^-2^), 2 g, 3 g, 4 g, 6 g, 8 g and 0 g (as a ‘no addition’ baseline). This range includes superficially high initial densities, as a trial experiment showed that an addition of 8 g of *H*. *diversicolor* was required to produce a density that was similar to the average density of *C*. *volutator* after 14 days. Each of the seven *H*. *diversicolor* density treatments was replicated three times, resulting in a total of 21 experimental plots. Given the presumed variation in emigration rate among plots, along with the spatial variation in *H*. *diversicolor* density at the study site, it was intended that the treatments would result in a smooth gradient from low to high density at sampling, rather than falling discretely into the initial density categories (i.e. *H*. *diversicolor* density at sampling would be treated as a quantitative explanatory variable in analyses; n = 21). Plots were distributed across the shore in three blocks at an elevation of 1 m above mean sea level. One block was laid each day from the 21^st^ to the 23^rd^ of June 2014. Each block contained one plot for each of the seven initial density treatments.

To provide a baseline from which to measure effects on ecosystem functioning, a corer (2 cm diameter, 2 cm depth) was used to collect a single surface sediment sample from the centre of each plot at the onset of the experiment (immediately prior to transplantation). Any macroinfaunal organisms contained within these samples were removed using forceps. Samples were then frozen at -15°C for later analysis of total carbon (C) and total nitrogen (N) content.

At the end of the experiment (12 days after transplanting *H*. *diversicolor*), individual surface sediment samples were again collected from the centre of each plot for analysis of total C and total N, as described above. The plots were then sampled in their entirety using a cylindrical corer (20 cm diameter, 20 cm depth). Samples were sieved through 0.5 mm mesh and the residue preserved in 70% ethanol. Retained fauna were identified to the lowest taxonomic level possible and their fresh biomasses weighed after soaking in freshwater and draining through filter paper for 30 minutes.

After freeze-drying the surface sediment samples, % total C and % total N were calculated using a Carlo Erba NC 2500 Elemental Analyser, calibrated using High Organic Standard OAS (Elemental Microanalysis Ltd) (C = 7.17% ± 0.09%; N = 0.57% ± 0.02%). The change in total C from the start to the end of the experiment was used as a measure of organic matter consumption. A greater net loss of total C (initial % total C–final % total C) was taken to indicate a greater consumption of organic matter. We therefore assumed that any deposition of C and any loss of C through non-consumptive processes was equivalent across all plots within a block. Change to the C:N ratio was used as an index of nutrient flux (*sensu* [41]). A greater increase in C:N during the experiment (final C:N–initial C:N) was taken to indicate a greater loss of total N relative to changes in total C (associated with organic matter consumption or deposition) and therefore a greater flux of nutrients into the overlying water.

### Data analysis

Statistical analyses were performed using R statistical software [43]. Data were analysed using general linear models. Assumptions of homoscedasticity and normality of residuals were checked by inspecting plots of residuals against fits and normal quantile plots, respectively. Data were ln-transformed, when required, to meet test assumptions. Null hypotheses were rejected at *p* < 0.05. The data for the microcosm and field experiments are provided in the Supporting Information (S1 File and S2 File, respectively).

#### Microcosm experiment

Variation in organic matter consumption (loss of mass of detrital *U*. *intestinalis*) and nutrient flux (DIN concentration) was analysed in relation to the densities of *C*. *volutator*, *H*. *diversicolor* and their interspecific interactions (*C*. volutator*H. *diversicolor*). The effects of density-dependent intraspecific interactions (*C*. *volutator*^2^ and *H*. *diversicolor*^2^) were also initially included in the models, in order to account for any influence that intraspecific competition may have on functioning, but were removed if statistically insignificant. Main effects (i.e. *C*. *volutator* and *H*. *diversicolor*) were tested by comparing models with and without the term of interest included. In accordance with the principal of marginality, higher-order terms pertaining to the term of interest (i.e. its square and interactions with other variables) were excluded from these models (see [44], section 6.8, *Marginality restrictions*). The effect of experimental block was included in all models. All terms except block were treated as quantitative variables.

As nutrients are remineralised by the break-down of organic matter [45], and these nutrients may be released into the overlying water when detritus is consumed by benthos (and therefore not be attributable to the burrowing activities of macroinfauna), the relationship between DIN concentration and the loss of mass of detrital *U*. *intestinalis* was tested, with block included in the model. Similarly, DIN concentration in the two control treatments was analysed to determine the influence of *U*. *intestinalis* addition and decomposition on nutrient flux in the absence of macroinfauna, with block again included in the model.

In one microcosm containing *U*. *intestinalis*, a mass of the detrital alga was found floating in the overlying water at the end of the experiment and had therefore not been available to benthic organisms. The resulting observation for organic matter consumption lay > 3 standard deviations from its expected value. The replicate was therefore removed from all analyses as the desired treatment had not been achieved. However, robust linear regression using MM-estimation (see [44], section 6.5; implemented in the R package **rlm**), without removing the outlying observation, produced almost identical coefficients to the least squares linear models, with the observation removed, for both organic matter consumption and nutrient flux.

Results are presented based on the densities of species recorded at sampling (i.e. final densities; see Table 1) to allow for consistency with field experiment analyses. Performing analyses using either the initial densities or the means of initial and final densities had little quantitative effect on the results (see S1 & S2 Tables, respectively).

**Table 1:** Linear model summary for the microcosm experiment analysis.

		Organic matter consumption		Nutrient flux	
	
Term	d.f.	F	*p*	F	*p*
**Block**	5,44	13.386	**< 0.0001**	3.698	**0.0070**
***Corophium volutator***	1,45	86.287	**< 0.0001**	13.161	**0.0007**
***Hediste diversicolor***	1,45	17.663	**0.0001**	17.639	**0.0001**
***C*. *volutator***^***2***^	1,42	2.859	0.0983	0.342	0.5616
***H*. *diversicolor***^***2***^	1,42	0.001	0.9824	1.104	0.2994
*C*. *volutator* *H. *diversicolor*	1,44	4.290	**0.0442**	6.208	**0.0166**

Effects of the densities of *Corophium volutator* and *Hediste diversicolor* on organic matter consumption (*Ulva intestinalis* consumed) and benthic-pelagic nutrient flux (ln-transformed dissolved inorganic nitrogen concentration) in laboratory microcosms. Squared density terms were initially included in the models to assess potential effects of intraspecific competition, but were removed because they were not statistically significant. The interaction term was also removed when testing the main effects. Significant *p*-values (< 0.05) are in bold.

#### Field experiment

**Fig 2:**
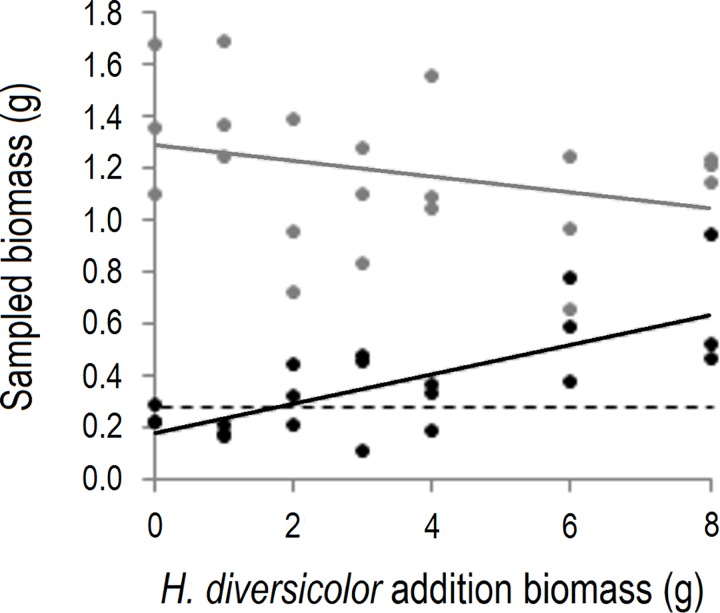
Transplants successfully raised the density of *H*. *diversicolor* beyond the ambient density. Variation in the sampled biomass (12 days after experiment initiation) of *Hediste diversicolor* (black; linear regression, R^2^ = 0.59) and *Corophium volutator* (grey; linear regression, R^2^ = 0.09) in relation to the addition biomass of *H*. *diversicolor* in field plots. The dashed line marks the maximum density of *H*. *diversicolor* recorded in plots with no experimental additions.

The densities of *H*. *diversicolor* and *C*. *volutator* at sampling were plotted against the transplanted density of *H*. *diversicolor*. This allowed assessment of whether the experimental treatment had: 1) raised *H*. *diversicolor* density beyond ambient levels, and 2) resulted in a shift in species dominance. To investigate whether *C*. *volutator* density declined in response to enhanced *H*. *diversicolor* density, the relationship between the densities of *C*. *volutator* and *H*. *diversicolor* at sampling was analysed, with block included in the model. Although the attempt to induce a shift in species dominance was unsuccessful, and there was no significant relationship between the densities of the two species (general linear model: F_1,18_ = 0.490; *p* = 0.4930), the density of *H*. *diversicolor* was successfully raised above ambient levels and the density of *C*. *volutator* was highly variable among plots (Fig 2). This allowed the influence of the densities of the two species (and their interactions) on function provision to be assessed.

Organic matter consumption (initial % total C–final % total C) and nutrient flux (final C:N–initial C:N) were analysed in relation to the densities of *C*. *volutator*, *H*. *diversicolor*, *C*. volutator*H. *diversicolor*, *C*. *volutator*^2^ and *H*. *diversicolor*^2^. Squared density terms were removed from the models if statistically insignificant. These analyses were performed as described as above for the microcosm experiment, except the densities of the other macroinfaunal species present at the study site (*Macoma balthica* and *Tubificoides* spp.) were also included in models to reduce residual variation. All terms except block were treated as quantitative variables.

## Results

### Microcosm experiment

**Fig 3:**
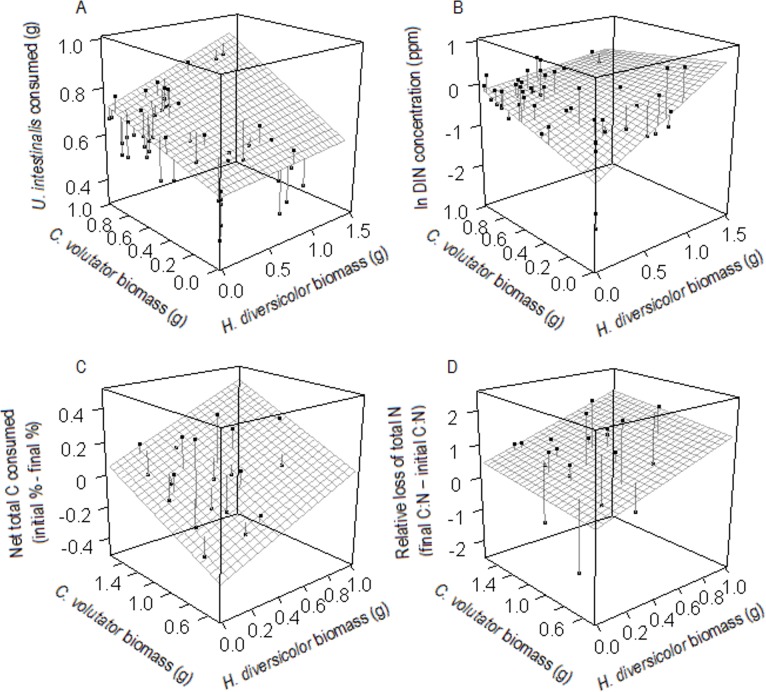
Ecological function provision vs. species densities in microcosm and field experiments. Variation in organic matter consumption and nutrient flux in relation to the densities of *Corophium volutator* and *Hediste diversicolor* in laboratory microcosms (A & B, respectively; DIN = dissolved inorganic nitrogen) and field plots (C & D, respectively). The 3D surfaces are based on the predicted values from the relevant linear models, with block set to ‘block 1’ (i.e. the first experimental run), other significant terms set to their means, and other non-significant terms excluded. The points represent the actual observations and the lines connecting the points to the 3D surface represent the size of the residuals. The interaction between *C*. *volutator* and *H*. *diversicolor* is included in plots A and B, as this term was significant (*p <* 0.05) in both of these models. The interaction was not included in plots C and D, as this term was not significant in these models (*p* > 0.05). The surface for D was plotted with respect to the densities of *C*. *volutator* and *H*. *diversicolor* to show the trend of the data; however, neither term was significant. Both terms plotted in C were significant.

Organic matter consumption (the loss of mass of detrital *U*. *intestinalis*) varied significantly in relation to *C*. volutator*H. *diversicolor* (Table 1). Increasing *C*. *volutator* density consistently had a positive effect on organic matter consumption, whereas the effect of increasing *H*. *diversicolor* density on organic matter consumption went from negligible to positive as the density of *C*. *volutator* increased (i.e. a positive interaction; Fig 3A). The effects of intraspecific interactions (*C*. *volutator*^*2*^ and *H*. *diversicolor*^2^) on organic matter consumption were not significant (*p* > 0.05; Table 1).

Nutrient flux (ln-transformed dissolved inorganic nitrogen (DIN) concentration) also varied significantly in relation to *C*. volutator*H. *diversicolor* (Table 1). Increasing the density of *C*. *volutator* or *H*. *diversicolor* had a positive effect on nutrient flux when the density of the other species was low, but these effects became negligible as the density of the other species increased (i.e. a negative interaction; Fig 3B). The effects of intraspecific interactions (*C*. *volutator*^2^ and *H*. *diversicolor*^2^) on nutrient flux were not significant (Table 1).

Nutrient flux (ln-transformed DIN concentration) did not vary significantly in relation to organic matter consumption (the loss of mass of detrital *U*. *intestinalis*) among microcosms (general linear model: F_1,46_ = 0.446; *p* = 0.5077). In control microcosms, DIN concentration was significantly lower when detrital *U*. *intestinalis* was added (ANOVA: F_1,4_ = 17.91; *p* = 0.0134; S1 Fig), indicating that, in the absence of macroinfauna, decomposition of *U*. *intestinalis* caused DIN to decline.

## Field experiment

Organic matter consumption (initial % total C–final % total C) increased significantly as the densities of *C*. *volutator* or *H*. *diversicolor* increased (Table 2; Fig 3C). *C*. volutator*H. *diversicolor*, *C*. *volutator*^*2*^ and *H*. *diversicolor*^2^ were not significant (Table 2).

**Table 2:** Linear model summary for the field experiment analysis.

		Organic matter consumption		Nutrient flux	
	
Term	d.f.	F	*p*	F	*p*
**Block**	2,14	0.090	0.7686	0.115	0.7391
***Corophium volutator***	1,15	6.346	**0.0236**	0.004	0.9500
***Hediste diversicolor***	1,15	6.942	**0.0188**	0.237	0.6332
***C*.*volutator***^***2***^	1,12	0.446	0.5167	0.011	0.9173
***H*. *diversicolor***^***2***^	1,12	0.165	0.6920	3.679	0.0792
*C*. *volutator* *H. *diversicolor*	1,14	1.264	0.2799	0.120	0.7340
***Macoma balthica***	1,14	0.003	0.9591	0.922	0.3532
***Tubificoides* spp.**	1,14	4.117	0.0619	0.518	0.4834

Effects of the densities of *Corophium volutator* and *Hediste diversicolor* on organic matter consumption (Total C before the experiment–total C after the experiment) and benthic-pelagic nutrient flux (C:N after the experiment–C:N before the experiment) in field plots. Squared density terms were initially included in the model to assess potential effects of intraspecific competition, but were removed because they were not statistically significant. The interaction term was also removed when testing the main effects. Significant *p*-values (< 0.05) are in bold.

Nutrient flux (final C:N–initial C:N) did not vary significantly with respect to the densities of *C*. *volutator*, *H*. *diversicolor*, *C*. volutator*H. *diversicolor*, *C*. *volutator*^*2*^ or *H*. *diversicolor*^2^ (Table 2; Fig 3D).

## Discussion

The present study found that concurrent changes to the densities of two common benthic invertebrates affected organic matter consumption and benthic-pelagic nutrient flux in a soft-sediment marine ecosystem. Our microcosm experiment indicated that interspecific interactions altered the effects that species had on both ecological functions. In field plots, species-specific effects on organic matter consumption appeared to be unaffected by interspecific interactions, whereas for nutrient flux no significant effects of species were observed. Our results reaffirm that the presence and densities of species can affect key ecological functions [11,28], but also suggest that these effects can vary depending on the composition of the assemblage with which they interact. An effect of interspecific interactions on functioning was, however, only detected under homogenous environmental conditions and not within a natural, heterogeneous ecosystem.

Within our two-species microcosm experiment, trait-based predictions of relationships between function provision and the density of each species (Fig 1, black lines) were only consistently supported when the density of the other species was low. As the density of the other species increased, interspecific interactions caused density-function relationships to depart from trait-based predictions in two ways: 1) a relationship emerged that was not predicted, e.g. *Hediste diversicolor* density had a positive effect on organic matter consumption only at high *Corophium volutator* densities, and 2) a relationship was not observed when one was predicted, e.g. the positive effect of *H*. *diversicolor* density on nutrient flux disappeared at high *C*. *volutator* densities (Fig 1, dashed lines). As postulated *a priori*, we suggest that the above interactions for both functions are explained by *C*. *volutator* causing *H*. *diversicolor* to switch from suspension-feeding to deposit-feeding (for review of feeding plasticity in *H*. *diversicolor*, see [24]) in response to reduced suspension-feeding efficiency due to sediment resuspension by *C*. *volutator* [38]. Other macroinfaunal species commonly used in marine ecosystem functioning experiments also cause sediment resuspension, e.g. the gastropod *Peringia ulvae* [46] and the bivalve *Macoma balthica* [47], which may partly explain why nutrient flux is often found to be highest in microcosms containing only *H*. *diversicolor* (e.g. [48,49]).

Aside from the proposed mechanism, there are other possible explanations for the significant interspecific interactions observed in the microcosm experiment. One possibility is that *H*. *diversicolor* caused *C*. *volutator* to increase its rate of deposit-feeding and reduce the rate at which it flushes its burrows. However, we are unaware of a mechanism through which this could occur. The activities of two species within the sediment might also have interacted to promote microbial decomposition of organic matter [50], which in the absence of macroinfauna caused DIN concentration to decline (S1 Fig) and thus may also explain the negative interaction observed for nutrient flux. Irrespective of the exact mechanism, the influence of interspecific interactions on ecosystem functioning can be considered as facilitative with regard to organic matter consumption and antagonistic with regard to nutrient flux. From an ecosystem functioning perspective, the nature of interspecific interactions (i.e. facilitation or antagonism) may therefore be dependent on the particular function considered.

Regarding our field experiment, there are various possible explanations for why results differ from those of the microcosm experiment and why no significant interspecific interactions were observed. First, we note that *C*. *volutator* density was higher than *H*. *diversicolor* density in almost all field plots (Fig 2). The chance of detecting the significant interaction observed for organic matter consumption within microcosms may therefore be comparatively small in the field, as the low densities of *C*. *volutator* at which *H*. *diversicolor* did not affect organic matter consumption within microcosms were not re-created in field plots. This may also explain why no significant effect of *H*. *diversicolor* density on nutrient flux was detected in the field, as this relationship was most positive when the density of *C*. *volutator* was low within microcosms (Fig 3B). The same may also apply regarding the lack of an effect of *C*. *volutator* on nutrient flux in the field; although there were some plots in which *H*. *diversicolor* biomass was close to zero (Fig 2), which is the condition under which a positive effect of *C*. *volutator* on nutrient flux would be expected to be strongest based on the microcosm experiment results (Fig 3B). If the above explanations are valid, then the results of the field experiment may essentially be consistent with those of the microcosm experiment.

With the possibility of congruence between microcosm and field experiment results considered, there are reasons why we might expect to observe different results under the different experimental contexts used in this study. For example, the effect of the abiotic environment on functioning may have masked biotic effects in the field [19]. Indeed, the effect of interspecific interactions on functioning appears to increase over time in natural ecosystems [31], and our experiment may have been of insufficient duration to detect their influence in the presence of environmental heterogeneity. Previous studies have reported that *C*. *volutator* is sensitive to oxygen depletion [51] and tends to move away from organically enriched patches of sediment, whereas *H*. *diversicolor* moves toward them [49,52]. It is therefore possible that confinement of species to enriched sediment within our microcosm experiment caused *C*. *voluator* to irrigate its burrows more regularly than usual in order to prevent hypoxia, thus resuspending more sediment and enhancing the effects of interspecific interactions compared to what would occur within a natural, open ecosystem. This conjecture assumes that the mechanism proposed to explain the observed interactions in the microcosm experiment was actually in effect. It is also possible that environmental heterogeneity in the field reduced the strength of interspecific interactions, as has been observed in fungal [16] and bacterial communities [18], or that interactions with other taxa present at the field site influenced the results. Finally, we reiterate that we used direct measurements of functions within microcosms and proxy measurements within field plots, which may have made us less likely to detect effects in the field. Therefore, while our results provide no evidence for interspecific interactions altering species-specific effects on functioning within a natural ecosystem, they should not be considered as an indication that such interactions were not occurring.

The species used in our study often dominate the infaunal biomass of intertidal mudflats of the northeast and northwest Atlantic, but can experience substantial changes to their densities in response to environmental fluctuations [24,32]. With mudflats typically supporting few species with which to compensate for population losses, our results may therefore have broad-scale implications for the functioning of this ecosystem. For example, our finding (from both microcosm and field experiments) that decreasing *C*. *volutator* density consistently leads to reduced organic matter consumption (Fig 3A and 3C) raises questions about the ability of mudflats to assimilate organic waste and remineralise the contained nutrients if populations of this species collapse. Such concerns are exacerbated by the fact that various historic populations of the species have collapsed in recent years, notably in the Wadden Sea [53,54]. If *C*. *volutator* is lost from an ecosystem, our microcosm experiment results suggest that a compensatory increase in *H*. *diversicolor* density may do little to mitigate the impact on organic matter consumption, as the positive effect of *H*. *diversicolor* on this function may disappear when *C*. *volutator* is absent (Fig 3A). In contrast, both of our experiments suggest that nutrient flux may be unaffected by a shift in the densities of the studied species. However, if populations of both species are reduced to low levels, then our microcosm experiment suggests that nutrient flux, and thus pelagic productivity, may consequently decline (Fig 3B). The risk of such collapses may be particularly great in the Northwest Atlantic, where populations of both species have recently been shown to consist of introduced subsets of European populations [55,56], whose low genetic diversity may make them highly vulnerable to environmental change [57,58].

To assess the impacts of ongoing biodiversity change requires that we can map specific changes in species assemblages to effects on the provision of ecological functions [20]. Biological traits (i.e. morphological, behavioural and life history characteristics) are the main medium through which individual species affect function provision, and therefore represent a tool through which the impact of biodiversity change on the functioning of ecosystems can be indirectly inferred [59,60]. As trait-based predictions of species density-function relationships were supported in our microcosm experiment only when the density of the other species was low, this suggests that interspecific interactions may impede our ability to accurately predict the impacts of biodiversity change in natural ecosystems based on simplistic assumptions about trait expression. Other recent studies have demonstrated that species can switch feeding modes depending on environmental conditions [61,62]. Methods used to infer functioning from traits, e.g. Biological Traits Analysis (BTA) [63,64], generally account for such trait plasticity using fuzzy-coding, whereby the degree to which species exhibit different strategies is approximated (e.g. 50% deposit-feeder, 50% suspension-feeder) [65]. Our results, and the results of the aforementioned studies, suggest that for BTA to predict even the direction of changes to function provision with confidence, biotic and abiotic context should be considered when classifying the traits of each constituent species. The possibility that changes to abiotic conditions may alter the nature of biotic interactions (i.e. facilitative vs antagonistic [21]), and that the activities of organisms can modify abiotic conditions [37], makes this task even more daunting. Nevertheless, it appears that an incorporation of species-species and species-environment interactions is required if we are to be able to accurately predict changes to functioning associated with ongoing biodiversity change.

## Supporting Information

S1 Fig The effect of detritus addition on nutrient levels in the absence of macroinfauna. Mean dissolved inorganic nitrogen (DIN) concentration (with 95% confidence intervals) in microcosms containing no macroinfauna with and without the addition of detrital *Ulva intestinalis*.
(TIF)

S1 File Microcosm experiment data. (XLSX)

S2 File Field experiment data. (XLSX)

S1 Table Linear model summary for the microcosm experiment analysis based on initial taxa densities. Effects of the densities of *Corophium volutator* and *Hediste diversicolor* on organic matter consumption (*Ulva intestinalis* consumed) and benthic-pelagic nutrient flux (ln-transformed dissolved inorganic nitrogen concentration) in laboratory microcosms based on initial taxa densities. Squared density terms were initially included in the model to assess potential effects of intraspecific competition, but were removed because they were not statistically significant. The interaction term was also removed when testing the main effects. Significant *p*-values (< 0.05) are in bold.
(TIF)

S2 Table Linear model summary for the microcosm experiment analysis based on the mean of initial and final taxa densities. Effects of the densities of *Corophium volutator* and *Hediste diversicolor* on organic matter consumption (*Ulva intestinalis* consumed) and benthic-pelagic nutrient flux (ln-transformed dissolved inorganic nitrogen concentration) in laboratory microcosms based on the mean of initial and final taxa densities. Squared density terms were initially included in the model to assess potential effects of intraspecific competition, but were removed because they were not statistically significant. The interaction term was also removed when testing the main effects. Significant *p*-values (< 0.05) are in bold.
(TIF)
