# Supplementary figures and images for: Species-Specific Effects on Ecosystem Functioning Can Be Altered by Interspecific Interactions
Source: PLoS One. 2016 Nov 3;11(11):e0165739. doi: 10.1371/journal.pone.0165739 (PMC5094745; doi:10.1371/journal.pone.0165739)

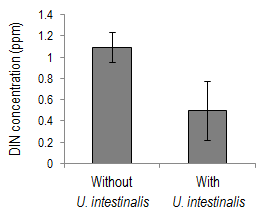

Supplement: S1 Fig — Mean dissolved inorganic nitrogen (DIN) concentration (with 95% confidence intervals) in microcosms containing no macroinfauna with and without the addition of detrital Ulva intestinalis. (TIF) [file pone.0165739.s001.tif]

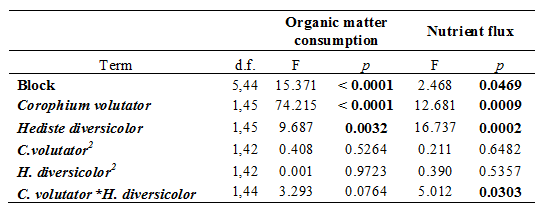

Supplement: S1 Table — Effects of the densities of Corophium volutator and Hediste diversicolor on organic matter consumption (Ulva intestinalis consumed) and benthic-pelagic nutrient flux (ln-transformed dissolved inorganic nitrogen concentration) in laboratory microcosms based on initial taxa densities. Squared density terms were initially included in the model to assess potential effects of intraspecific competition, but were removed because they were not statistically significant. The interaction term was also removed when testing the main effects. Significant p-values (< 0.05) are in bold. (TIF) [file pone.0165739.s004.tif]

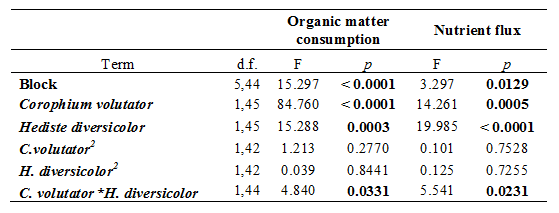

Supplement: S2 Table — Effects of the densities of Corophium volutator and Hediste diversicolor on organic matter consumption (Ulva intestinalis consumed) and benthic-pelagic nutrient flux (ln-transformed dissolved inorganic nitrogen concentration) in laboratory microcosms based on the mean of initial and final taxa densities. Squared density terms were initially included in the model to assess potential effects of intraspecific competition, but were removed because they were not statistically significant. The interaction term was also removed when testing the main effects. Significant p-values (< 0.05) are in bold. (TIF) [file pone.0165739.s005.tif]
